# Antimicrobial activity and toxicity of glass ionomer cement containing an essential oil

**DOI:** 10.1590/1414-431X20209468

**Published:** 2020-10-30

**Authors:** J.M.F.F. Nunes, I.A.P. Farias, C.A. Vieira, T.M. Ribeiro, F.C. Sampaio, V.A. Menezes

**Affiliations:** 1 Laboratório de Biologia Bucal, Centro de Ciências da Saúde, Universidade Federal da Paraíba, João Pessoa, PB, Brasil; 2 Departamento de Odontologia, Faculdade de Odontologia, Universidade de Pernambuco, Camaragibe, PE, Brasil

**Keywords:** Glass ionomer cements, Biofilms, Thymol, Streptococcus mutans, Anti-infective agents

## Abstract

The aim of this study was to evaluate the antimicrobial activity and toxicity of glass ionomer cement (GIC) modified with 5-methyl-2-(1-methylethyl)phenol (thymol) against *Streptococcus mutans in silico* and *in vitro*. The antimicrobial activity of thymol on GIC modified with concentrations of 2% (GIC-2) and 4% (GIC-4) was evaluated in a model of planktonic cell biofilm using agar diffusion test, minimum inhibitory concentration (MIC), minimum bactericidal concentration (MBC), dynamic biofilm (continuous flow cell parallel), and bacterial kinetics. Conventional GIC (GIC-0) was used as a control. Thymol toxicity was evaluated in *Artemia salina* and *in silico* using Osiris^®^ software. Differences between groups were estimated by analysis of variance, followed by Tukey *post hoc* test, with a 5% significance level. The results of the agar diffusion test between groups were not significantly different (P≥0.05). Thymol had potential bacteriostatic and bactericidal activity against *Streptococcus mutans* with respect to planktonic growth, with MIC of 100 µg/mL and MBC of 400 µg/mL. The groups GIC-0, GIC-2, and GIC-4 reduced the biofilm by approximately 10, 85, and 95%, respectively. Bacterial kinetics showed efficiency of the modified GICs for up to 96 h. GIC with thymol was effective against *S. mutans*, with significant inhibition of the biofilms. Analyses *in silico* and using *Artemia salina* resulted in no relevant toxicity, suggesting potential for use in humans. GIC-2 was effective against *S. mutans* biofilm, with decreased cell viability.

## Introduction

Among the restorative materials used in dentistry, glass ionomer cement (GIC) is widely used because of its biocompatibility, antimicrobial action, adhesion to tooth structure, and release of fluoride (1,2). After a period of time from the completion of a dental restoration, biofilm formation occurs on the GIC surface with subsequent degradation of the material, which limits clinical performance over time. The reduced longevity of dental materials (3) may be responsible for half of the restoration failures that occur over the first 10 years (4).

Various studies have combined antimicrobial agents with restorative materials (especially with GIC) in an attempt to reduce the formation of biofilm and prevent the occurrence of secondary caries, thus improving antimicrobial activity while enhancing clinical efficacy (5–7). Conventional GIC has been combined with antibiotics (1.5, 3, and 4.5% doxycycline hyclate and 1.5 and 3% 1:1:1 ciprofloxacin:metronidazole:minocycline) and exhibited excellent antimicrobial properties (8,9). When associated with cetylpyridinium chloride, cetrimide, ethanolic extracts of propolis, and benzalkonium chloride, GIC presents antimicrobial activity against bacteria of the genera *Streptococcus*, *Lactobacillus*, and *Actinomyces*, showing significant inhibition of bacterial growth (6,7,9,10). The *Streptococcus* genus has been the subject of research because of its importance to the cariogenic process, and *Streptococcus mutans* is the most studied species of this genus because of its relationship with the onset and microbiological predictors of dental caries (11–13).

Among the surveyed antimicrobials used in dentistry, essential oils (EOs) have been regarded as promising molecules. EOs are volatile aromatic liquids and complex plant materials known for their medicinal properties, such as analgesia, sedation, anti-inflammatory, spasmolytic, and local anesthetic activities. The main groups of EOs are terpenes, such as carvacrol and thymol, and terpenoids, comprised of menthol and other aromatic and aliphatic components, such as eugenol and chavicol (14–18). Regarding toxicity, since these compounds are lipophilic, EOs readily cross plasma membranes and cell walls, and they may disrupt essential components of cells and bacteria. Moreover, in contact with EOs, the cytoplasm may coagulate and cause damage to lipids and proteins (16 [Bibr B17],^19^,^20^).

The antimicrobial activity of thymol (5-methyl-2-(1-methylethyl)phenol) alone or in combination with other EOs has been suggested by some studies (21). The incorporation of thymol, boric acid, and chloroxylenol at concentrations of 2 and 5% into GIC powder in evaluations of antimicrobial activity against *Streptococcus mutans* resulted in increases in inhibition compared with GIC alone. The compound thymol showed maximal inhibition (7).

From this perspective, this study aimed to evaluate the *in vitro* antimicrobial activity of a GIC containing an EO from the terpene group (thymol) in a model of planktonic cells and *S. mutans* biofilm, to verify the toxicity of thymol by applying an *in silico* model, and the toxicity of the restorative material containing thymol *in vivo* with brine shrimp. The null hypotheses of the study were that: 1) the incorporation of a terpene group (thymol) into GIC causes no change in the antimicrobial/antibiofilm capabilities and 2) thymol has no toxicity when applied *in silico* and *in vitro* models.

## Material and Methods

### Experimental design

This study experimentally assessed the *in vitro* antimicrobial activity of thymol-modified GIC and the *in vitro* toxicity of thymol. In addition, an *in silico* study was carried out to predict the chemical chronic toxicity profile.

### Preparation of thymol-incorporated GIC

In the experimental groups, pure essential thymol oil (>98% purity; batch number: 604-032-00; molecular formula: C_10_H_14_O; molecular weight: 150.22 g/mol; Sigma-Aldrich, Brazil) was weighed on an analytical balance and added by slight manual agitation to the powder of control GIC Maxxion™ (FGM, Brazil) at concentrations of 2% w/w (GIC-2) and 4% w/w (GIC-4). The control group was GIC without essential oil incorporated (GIC-0) (2,8–10).

The thymol concentration was based on: a) a general consensus that the adhesion of microbes to a surface favors bacterial metabolism and growth, and antimicrobials must have higher concentrations to be effective; b) thymol will be present in the surface and not in the liquid phase, and c) previous studies showing that an increase of substances in the GIC above 5% might reduce physical properties of this dental material.

### Agar diffusion test

The culture medium brain heart infusion agar (BHI agar^®^ (Difco, USA)) was used, and an aliquot of 400 µL of the bacterial strain (*Streptococcus mutans* ATCC 25175, USA) stock was transferred to vials containing 6 mL of BHI broth and was incubated in microaerophilic conditions for 24 h at 37°C. The microorganisms grown overnight were centrifuged at 4193 *g*, at room temperature for 10 minutes (Quimis, Diadema, Brazil), followed by removal of the supernatant, suspension in 0.9% saline, and standardization for absorbance in a spectrophotometer. The 0.135 absorbance value was equivalent to 0.5 on the McFarland scale (approximately 1.5×10^8^ cells/mL, 10^8^ CFU/mL).

The plates were seeded with 1000 µL of the bacterial inoculum standardized according to the McFarland scale, and the inoculum was spread evenly over the agar surface with the aid of a disposable Drigalski spatula (Olen, Kasvi, Brazil). After drying, sterile forceps and clinical steel cylinders (8.0×10.0 mm with a 6.0-mm internal diameter) were used to make 3 spaced wells to prevent halo fusion, which were filled to the height of the agar (22). The GIC groups were handled in glass plates with a sterile spatula, and a same volume of material was placed in each well.

After curing the material, the plate was covered and incubated for 24 h at 37°C. The reading of inhibition zones (cm) was performed with a precision of 0.05 cm using calipers. The shortest distance (cm) from the outer edge of the well to the starting point of microbial growth was regarded as the inhibition zone. The antimicrobial activity of each product was defined as the average of the inhibition halo values that were greater than or equal to 2 cm.

### Microdilution test for planktonic cells

The minimum inhibitory concentration (MIC) and minimum bactericidal concentration (MBC) were tested in an experimental model of dilution in liquid phase in microdilution plates using a 96-well U-bottom plates (23–25). Suspensions of bacteria were prepared with densities equal to that of a 0.5 McFarland scale turbidity standard. The MIC determination was made using a fluorescence kit used to determine bacterial viability (LIVE/DEAD^®^ BacLight™ bacterial viability kit L13152, Molecular Probes, USA) consisting of a 1:1 proportion of SYTO^®^ 9 and propidium iodide.

Aliquots of 30 µL were pipetted and dispensed into the wells of the microplate specific for fluorescence (UV-Star^®^ 96-wells, Greiner Bio-One North America, Inc., USA). Dye was added in an equal volume, and the microplate was allowed to stand for 10 min before measurement. Fluorescent intensity was measured using a multi-mode microplate reader (FLUORStar Optima, BMGLab Tech, Germany). Before measurement of the samples, a standard curve of cell viability was generated from 5 points in which reference values were obtained as percentages of live and dead bacteria (26).

### Dynamic biofilm model

The model consisted of the formation of an *in vitro* mono-species biofilm over 12 h using a dynamic biofilm (continuous flow cell in parallel - CFCP), specimens of the material, modified artificial saliva, and microorganisms (27).

Test specimens were prepared in a polypropylene matrix with an 8-mm diameter that was 2 mm in height. The studied materials (GIC-0, GIC-2, and GIC-4) were mixed in a powder/liquid proportion of 1:1, according to the manufacturer's guidelines (FGM, Brazil).

Before the experiment, to allow the formation of a conditioning film on the specimens, 1.5 mL of artificial saliva was added. The specimens were immersed in saliva and incubated for 5 min at room temperature. They were then carefully removed and inserted into the CFCP (Model FC 271, BioSurface Technologies Corporation, USA). The CFCP has two entries, and each entry has two circular recesses to accommodate the samples, which allows biofilm formation in duplicate. A peristaltic pump connected to the CFCP insures a continuous recycling stream of 3 L of modified artificial saliva (MAS) at a speed of 1.2 mL/min, inoculated with *Streptococcus mutans* in a ratio of 6 mL of the McFarland inoculum for each liter of artificial saliva. During the 12 h experimental test, the inoculum and MAS mixture were kept under magnetic stirring in an oven at 37°C.

The composition of the artificial saliva made in 1000 mL of deionized water was 1 g/L meat extract, 2 g/L yeast extract, 5 g/L protease peptone, 2.5 g/L gastric mucin pork type III, 0.2 g/L sodium chloride, 0.2 g/L potassium chloride, 0.3 g/L calcium chloride, 1.25 mL/L 40% urea solution, and BHI broth supplemented with 15% sucrose at a ratio of 60:40 (v:v). The urea solution was sterilized and filtered through a 0.2 µM filter. The pH of the saliva was adjusted to 6.9 after autoclave sterilization by the addition of a 40% urea solution.

Valves and syringes with air filters for exhaust bubbles were connected between the peristaltic pump and CFCP. After the trial period of 12 h, the peristaltic pump was turned off and the flow of MAS and inoculum was interrupted. The experiment was performed in triplicate for each pair of samples from each experimental group (GIC-0, GIC-2, and GIC-4).

For the biofilm treatment, the specimens were removed from the CFCP, immersed in autoclaved polypropylene tubes containing 5 mL of 0.9% saline, and shaken for 30 s on a shaking table. The specimens were then transferred to a Falcon-type tube containing 1 mL of 0.9% saline to collect the bacteria adhered to the biofilm using ultrasound (Unique, Brazil) for 2 min. The specimens were discarded, and the liquid containing the bacterial mass was transferred to Eppendorf tubes and centrifuged at 6037 *g*, for 10 min at room temperature (Quimis, Diadema, Brazil). The supernatant was discarded and 1 mL of 0.9% saline was added to the tubes.

The bacterial solution was homogenized by vortexing for 1 min and the absorbance was recorded spectrophotometrically, as previously described. The cell viability of all samples was also verified by fluorescence assays, as described above.

### Bacterial kinetics

A bacterial inoculum of *Streptococcus mutans* ATCC 25175 was standardized according to the McFarland scale and used. Specimens of the three experimental groups were suspended with dental floss perpendicularly to the lids of 24-well cell culture plates. They were immersed in BHI broth solution and standardized to the bacterial inoculum on the McFarland scale at a ratio of 8:2 and incubated for a period of 96 h.

At each reading stage, the specimens were subjected to a dip of 10 s in 5 mL of 0.9% saline to remove the free cells, followed by a second dip in 1 mL of 0.9% saline in polypropylene tubes. The samples were subjected to ultrasound for 2 min for removal of biofilm aggregate on the surface, which was then analyzed by fluorescence for the quantification of viable cells.

### Toxicity tests with *Artemia salina* Leach

*Artemia salina* cytotoxicity assays were based on the method described previously (28) with some modifications. A glass tank 25-cm long, 25-cm wide, and 25-cm high was used and illuminated with an LED lamp. For the preparation of synthetic seawater to 3.3%, sea salt (Sigma-Aldrich, Merck, Brazil) was used and the water was oxygenated with a common air pump. *A. salina* cysts were maintained until hatching of the nauplii (for 48 h), which were collected with a pointing device and an automatic pipette.

Thymol solutions were prepared at concentrations of 1000, 100, 10, 1, and 0.1 µg/mL. These values provide a range of 10^-1^ to 10^-25^ percentage of thymol. Each of the solutions of the series received 10 nauplii. Each dose was performed in quadruplicate, and for each test, a negative control tube was prepared containing 10 mL of brine (alone) and 10 newly hatched nauplii. After 24 h, the number of dead nauplii was counted in each tube.

Toxicity was classified as high, moderate, low, and non-toxic when death was obtained (characterized by cessation of nauplii swimming activity) at respective concentrations <100 μg/mL (high), 100 to 500 μg/mL (moderate), between >500 and <1000 μg/mL (low), and above 1000 μg/mL (non-toxic) (28).

### *in silico* analysis

The toxicity risk assessment of the studied molecules was determined *in silico* using Osiris^®^ Property Explorer software (2001-2014, Organic Chemistry Portal, Switzerland), a tool that usually indicates the presence of molecules responsible for irritant, mutagenic, tumorigenic, reproductive, or teratogenic effects. The software is based on a collection of 5,300 molecular fragments obtained from 3,300 commercial drugs and from 15,000 Fluka collection compounds, which are not commercial drugs.

The Molinspiration^®^ bioactivity score v2011.06 (Molinspiration of Products and Services, Slovak Republic) was also used, which uses sophisticated Bayesian statistics to compare structures of representative ligands active on the particular target with inactive molecule structures and to identify typical sub-structural characteristics for active molecules. The system searches for possible interactions with G proteins, ion channel modulation, kinase inhibitors, nuclear receptors, protease inhibitors, and other enzyme-based targets. The higher the score, the greater the probability of the molecule being active.

### Statistical analysis

Data are reported as means and standard deviation. Differences between groups were estimated by analysis of variance (ANOVA), followed by Tukey’s *post hoc* test, considering the halo of the inhibition zones and the viability of the cells in the dynamic biofilm model. A variance analysis using two criteria (time and concentration of material) was performed for the bacterial kinetic test, with a 5% significance level, using IBM SPSS Statistics (USA), version 21.0. The median lethal concentration (LC_50_) was determined by analyzing the data using linear regression analysis.

## Results

The agar diffusion test showed similar antimicrobial activity between groups, but the average of the halos of the inhibition zones was less than 2 cm for all groups (P>0.05). The mean and standard deviation of the halo diameters formed for the GIC-0, GIC-2, and GIC-4 groups were 1.50 (±0.44), 1.89 (±0.11), and 1.70 (±0.15) mm, respectively.

The molecule 5-methyl-2-(1-methylethyl)phenol (thymol) showed bactericidal and bacteriostatic activity against the planktonic cells of *S. mutans* with an MIC of 100 μg/mL and an MBC of 400 μg/mL. The control group used chlorhexidine gluconate, which showed an MIC and an MBC of 2.3 and 9.4 μg/mL, respectively.

**Figure 1:**
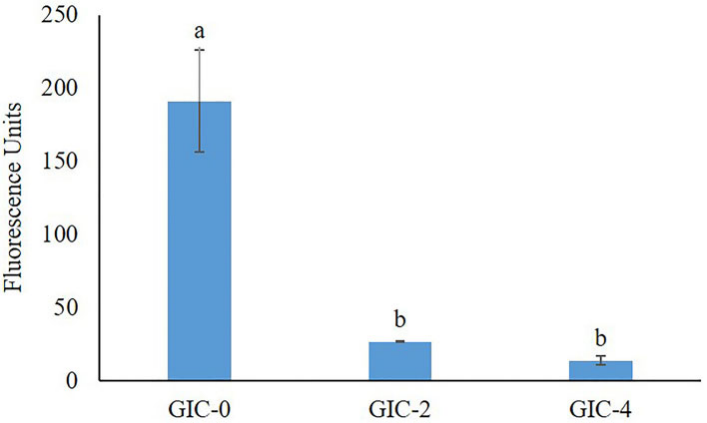
Growth of *S. mutans* in dynamic biofilms on glass ionomer cement (GIC) modified with thymol at different concentrations: 0, 2, and 4%. Data are reported as mean±SD. Different letters indicate significant differences between groups (P<0.001, ANOVA and Tukey’s test).

The dynamic biofilm quantitation test showed viable cells. The fluorescence results for GIC-0, GIC-2, and GIC-4 had means (±SD) of 191.33 (±5.05), 26.80 (±0.45), and 14.03 (±2.96), respectively (Figure 1). Statistically significant differences were observed between the modified cements and the negative control (P<0.001). A statistically significant difference was not observed between GIC-2 and GIC-4 (P=0.73). The GIC-0, GIC-2, and GIC-4 groups reduced the biofilms by approximately 10, 85, and 95%, respectively.

**Figure 2:**
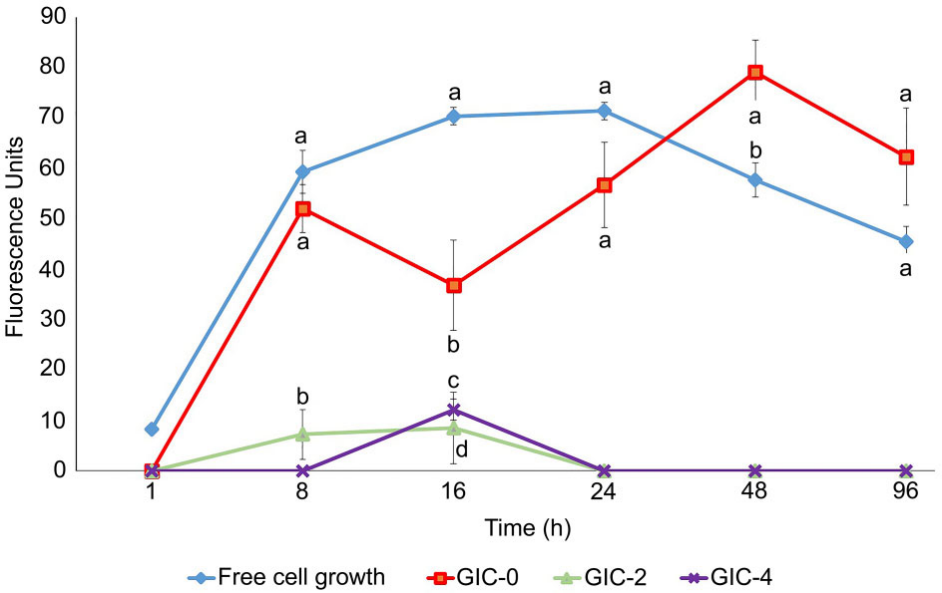
Growth of *S. mutans* as a function of time for glass ionomer cement (GIC) modified with thymol at different concentrations: 0, 2, and 4%. Data are reported as mean±SD. Different letters indicate significant differences between groups (P<0.05, ANOVA and Tukey’s test).

The effects of GICs modified with thymol on *S. mutans* growth were evaluated as a function of time. Testing revealed that thymol was able to markedly interfere with the logarithmic phase of the bacteria compared to the positive and negative controls (Figure 2). Within 8 h, the mean (±SD) of bacteria by fluorescence of the GIC-0, GIC-2, GIC-4, and negative control groups was 51.98 (±4.74), 7.20 (±4.93), 0.00 (±0.00), and 59.32 (±4.27), respectively. A statistically significant difference was observed between the GIC-2 group and the positive and negative controls (P<0.001 for both comparisons). A statistically significant difference was not observed between GIC-0 and the negative control (P=0.21). The bacterial kinetics test showed great sensitivity of *S. mutans* to the modified GICs, with the effect lasting throughout the 96 h of exposure.

The toxicity test against *Artemia salina* Leach revealed that thymol has low toxicity, with dead nauplii at concentrations equal to or above 1000 μg/mL (100% nauplii death). There was 30% of nauplii death at 100 μg/mL. The concentration required to induce a 50% lethal effect on nauplii survival (LC_50_) was achieved at 471.58 μg/mL thymol.

The *in silico* analysis of thymol with the online tool Osiris^®^ Property Explorer revealed possible mutagenic and reproductive effects but low irritant or tumorigenic risks. Thymol also scored low for TPSA (Topological Polar Surface Area) suggesting low permeability.

The Molinspiration^®^ tool bioactivity score (v2011.060) indicated a great likelihood for thymol to act as an ion channel modulator and as an enzyme inhibitor (29).

## Discussion

Thymol-modified GIC has been tested previously and showed antimicrobial activity against *S. mutans* (30,31), *Candida albicans*, *S. mitis*, *S. salivarius*, and *S. sanguis*(30). However, the agar diffusion test presented false negatives (halos of inhibition smaller than 2 cm), confirming some studies that have also shown no activity with conventional glass ionomer cement (10,32) for the inherent characteristic of restorative materials (10), as the diffusion test was originally designed for antibiotics. The factors that influence the specificity of the method, such as the molecular diffusion rate in agar, lack of knowledge on the interaction between the material tested and the agar components, the fact that plate chemistry is different from body chemistry, and the different influence of pH on planktonic cells compared to dynamic biofilms, must be considered (33).

The dynamic biofilm test showed that thymol-modified GIC significantly reduced viable cells at the 2% thymol concentration. The kinetics test revealed lasting effects for over 96 h of exposure. The amount of viable *S. mutans* cells was higher than that from the conventional GIC, which was unable to influence the growth of biofilm over this length of time.

One limitation of this study is the fact that the challenges of *in vivo* biofilm formation are continuous over the life of the restoration, while the GIC-2 and GIC-4 evaluation time in this model was 48 h. Although knowledge of the thymol releasing behavior after 48 h is limited, in the oral environment, frequent thymol release is assumed, due to the high solubility of GICs in general.

In addition, the antibacterial action of conventional GIC may be linked to the continuous fluoride release rate (8,34,35). It is known that the released fluoride slows down the progression of caries lesions on tooth surfaces adjacent to dental materials. The release of fluoride from GICs contributes to controlling biofilm formation, particularly in its early phases. Thymol essential oil is incorporated into the entire body of the material so that mass loss by abrasion or dissolution facilitates its release. Considering the dentin-restoration interface, one cannot rule out the potential for the anti-inflammatory and anesthetic effects of thymol. Thus, the biocompatibility of GIC might be enhanced. However, the anti-caries effect of fluoride-releasing materials is still not supported by clinical evidence, and in addition, these effects can be overcome by fluoride delivered from dentifrices. This effect has been clearly shown by *in vitro* and *in situ* studies but not in randomized clinical trials (34,35).

The biofilm model experiments showed a significant ability of thymol to reduce biofilm adherence to the material surface at both concentrations tested. This result is interesting considering that microbial biofilms can be up to one thousand times more resistant to antibacterial compounds than planktonic cells. On the surface of the tooth and GIC containing thymol, biofilm formation would develop differently, considering that it is in biofilm that cellular interactions take place (physical and nutritional synergistic association, cell-to-cell communication, and gene transfer) (36,37). Hence a greater concentration of the antimicrobial agent on biofilm is needed compared to planktonic cells.

A limitation of this study is that it evaluated a mono-species biofilm. However, the increased complexity of a multi-species biofilm might hinder fluorescence evaluations (38). While the *S. mutans* dynamic biofilm model does not mimic the complex microbiota found in dental biofilms, the qualitative and quantitative model used in this work is interesting because it verifies cell viability and the cariogenic process of biofilms.

The kinetics study and biofilm growth results indicated that thymol is a consistent modulator of biofilm evolution on the surface of the test specimen/material. This fact supports the execution of further tests with thymol at lower concentrations to better preserve the mechanical properties of the material while retaining the potential for incorporating antimicrobial activity.

It is interesting to note that the type(s) of bacteria defines both antimicrobial resistance and the intrinsic resistance of the biofilm. In this study, a biofilm with an ATCC strain was chosen to minimize the effects of additional resistance coming from wild strains. For this reason, biofilm designs must also consider that the antimicrobial effect of the molecules tested might be lower than expected when the same tests are repeated in wild strain biofilms.

The low toxicity of the cement (as per toxicological testing) indicated that thymol is safe for improving restorative materials. Similar toxicological features have been found for plants (with thymol as their active principle) in animal models (39).

*In silico* analyses promote alternative ways by providing new analysis conditions; the use of computational tools can target the efficiencies of molecules against certain microorganisms and against systemic effects that are likely to occur when in contact with the human body. Low permeability is important for the molecule's access to the rest of the body. If swallowed, the molecule has a small chance of interfering with the body at the systemic level. This parameter is important because it is related to human intestinal absorption and penetration through the blood brain barrier (29,40). These analyses ranked thymol molecules as safe, although their concentrations require more attention. The possible role of thymol as an ion channel modulator and as an enzyme inhibitor is also encouraging because these are some of the most sought-after antimicrobial characteristics.

It is noteworthy that the LD_50_ of thymol is high (39) and therefore reveals tolerable thymol toxicity. This result indicated that the use of thymol incorporated into GIC is safe because the possible swallowing of restorative material would be significantly below a toxic amount and the amount of thymol incorporated into GIC is well below toxic values for humans. Furthermore, the amount of essential oil used for restoration is almost the same as that indicated for the preservation of foods that are consumed daily.

Generally, the results of this study suggested that incorporation of 2% thymol into GIC results in a restorative dental material with antimicrobial potential against *S. mutans*, the primary pathogen involved in the etiology of dental caries. The effect does not compromise the mechanical properties of the material (data not shown), since the incorporation of concentrations above 3% of antimicrobial agents in the conventional GIC caused significant changes in the mechanical properties (32). In addition, this incorporation can be performed safely, without deleterious effects from a toxicological point of view.

Further studies are required to test the clinical efficacy of the use of thymol-modified GIC in restorative procedures. In addition, the effects on dental biofilm and physical properties (for example, bond strength, fluoride release, flexure, compression, and tensile strength) must be verified in future studies.

### Conclusions

Based on the results of this study and considering the limitations, the inclusion of thymol into GIC maximizes the antibacterial effect of GIC without jeopardizing the important property of low toxicity, with low irritant or tumorigenic risks and low permeability, making use by humans possible with no apparent health risks. It may be concluded that the incorporation of 2% thymol into GIC showed bactericidal and bacteriostatic activity and was effective against *S. mutans* biofilm, with decreased cell viability in the biofilm on the material surface.
